# Aortic Valve-in-Valve Procedures: Challenges and Future Directions

**DOI:** 10.3390/jcm13164723

**Published:** 2024-08-12

**Authors:** Davide Cao, Stefano Albani, Emmanuel Gall, Thomas Hovasse, Thierry Unterseeh, Patrick Seknadji, Stéphane Champagne, Philippe Garot, Neila Sayah, Mariama Akodad

**Affiliations:** 1 Ramsay Générale de Santé, Institut Cardiovasculaire Paris Sud, 6 Avenue du Noyer Lambert, 91100 Massy, France; 2 Department of Biomedical Sciences, Humanitas University, 20072 Pieve Emanuele, Italy; 3 Division of Cardiology, U. Parini Hospital, 11100 Aosta, Italy; 4 Department of Cardiology, University Hospital of Lariboisiere, Université Paris-Cité, (Assistance Publique des Hôpitaux de Paris, AP-HP), 75010 Paris, France; 5 Inserm MASCOT—UMRS 942, University Hospital of Lariboisiere, 75010 Paris, France; 6 MIRACL.ai Laboratory, Multimodality Imaging for Research and Artificial Intelligence Core Laboratory, University Hospital of Lariboisiere (AP-HP), 75010 Paris, France

**Keywords:** coronary obstruction, valve-in-valve, TAVR

## Abstract

Aortic valve-in-valve (ViV) procedures are increasingly performed for the treatment of surgical bioprosthetic valve failure in patients at intermediate to high surgical risk. Although ViV procedures offer indisputable benefits in terms of procedural time, in-hospital length of stay, and avoidance of surgical complications, they also present unique challenges. Growing awareness of the technical difficulties and potential threats associated with ViV procedures mandates careful preprocedural planning. This review article offers an overview of the current state-of-the-art ViV procedures, with focus on patient and device selection, procedural planning, potential complications, and long-term outcomes. Finally, it discusses current research efforts and future directions aimed at improving ViV procedural success and patient outcomes.

## 1. Introduction

For decades, surgical aortic valve replacement has been the only treatment option for severe aortic stenosis until transcatheter techniques were developed [1,2]. Millions of patients worldwide require a prosthetic valve. Although mechanical valves are preferable in younger individuals owing to their longer durability, [3] biological prostheses are often perceived as less burdensome to patients due to non-obligatory oral anticoagulation [4,5]. Unfortunately, bioprosthetic valves suffer from higher odds of structural degeneration, which may eventually require reintervention after a variable time span. The rate of reoperation after bioprosthetic aortic valve placement approximates 30% at 15 years [6]. Frequently, in those patients, a redo surgery is considered at prohibitive risk of procedural complications [7].

Aortic ViV interventions consist of implanting a transcatheter heart valve (THV) within a previous, degenerated aortic bioprosthetic valve. The limited valve durability, along with expanding treatment indications and options [8], and the ever-increasing life expectancy of the general population has led, in the transcatheter era, to the spread of ViV procedures, particularly among elderly and high-risk individuals [9]. Despite the excellent outcomes [10], as any invasive procedure, ViV may be affected by major, life-threatening complications that should be anticipated during procedural planning and prevented at the time of the intervention.

## 2. Patient Selection and Outcomes

The European and American guidelines consider ViV an option for treating degenerated bioprosthetic valves in symptomatic patients at intermediate or high surgical risk and suggest that ViV is reasonable when performed at high-volume valve centers [11,12]. The evidence in support of redo surgery, achieved by fully replacing the degenerated valve, derives from studies performed mainly in younger and low-risk patients [13,14]. However, recent evidence supports the safety and efficacy profile of ViV procedures (Table 1), even when compared with redo surgical aortic valve replacement [15,16,17,18]. Meta-analyses have shown that among patients undergoing ViV, such intervention provides better outcomes compared to redo surgery in terms of perioperative morbidity and length of hospital stay [10,19,20]. Moreover, compared with redo surgery, ViV has been associated with lower short-term mortality (2.8% vs. 5.0% *p* = 0.02) and improved valve hemodynamics [10]. No significant differences between the two approaches have been noted with respect to stroke, myocardial infarction, or pacemaker implantation. Nonetheless, ViV carries a substantial risk of paravalvular leak (PVL) (risk ratio 4.18, *p* = 0.003) and prosthesis–patient mismatch (PPM) (risk ratio, 3.12, *p* < 0.001) [10].

Notably, the advantage of ViV over redo surgery may not be consistent over time. In a large cohort study of 6769 patients from the U.S. national readmission database, ViV was associated with lower in-hospital mortality and a higher rate of 30-day and 6-month all-cause readmission compared with redo surgery [21]. Another propensity score analysis of 375 patient pairs found that ViV was associated with a lower risk of postprocedural complications (major bleeding, 2.4% vs. 5.1%, *p* =  0.05; acute kidney failure, 1.3% vs. 7.2%, *p* < 0.001; new pacemaker implantation, 3.5% vs. 10.9%, *p* < 0.001), faster recovery times, and a similar 2-year mortality (hazard ratio, 1.03). Between 2 and 5 years, however, ViV patients experience higher mortality (hazard ratio, 2.97) and heart failure hospitalizations (hazard ratio, 3.81). A pooled analysis of Kaplan–Meier-derived data including 16 retrospective studies and 4373 patients highlighted a lower risk of mortality with ViV in the first 6 months (hazard ratio, 0.58, *p* < 0.001). However, this early survival benefit reversed over time, with redo surgery offering better survival at a later stage, beyond 6 months (hazard ratio, 1.92, *p* < 0.001) [22].

Owing to its retrospective nature, most of the available evidence suffers from a substantial risk of bias, with ViV patients being more often older, with greater burden of comorbidities and smaller aortic valves [22]. Dedicated randomized trials are therefore needed to clearly define treatment indications in case of failed aortic bioprostheses because of structural valve deterioration.

## 3. Tools for Procedural Planning

Transthoracic echocardiography should be routinely performed as the first step to confirm the diagnosis and rule out other conditions such as prosthetic valve endocarditis or thrombosis. If this is inconclusive, transesophageal echocardiography can prompt a definitive diagnosis while also providing higher accuracy for detecting infective endocarditis and paravalvular regurgitation.

The careful collection of information regarding the degenerated prosthetic valve (manufacturer, model, size), previous procedural complications, and details about the previous surgery (e.g., root replacement, coronary reimplantation, bypass grafts) is paramount for an accurate procedural planning.

Coronary angiography to exclude the presence of obstructive coronary stenoses is generally recommended and may help to clarify the interaction between the surgical bioprosthetic valve and the coronary arteries.

Non-invasive multidetector computed tomography (MDCT) coronary angiography during standard ViV work-up may be considered in patients with a low pre-test probability of coronary artery disease. Cardiac MDCT is indeed a cornerstone for the procedural planning of ViV interventions providing detailed information regarding annular dimensions, valve type, and size; prosthetic valve thrombosis or calcifications; peripheral vascular access anatomy, including lumen size, calcium, and tortuosity; and risk of complications, such as for PPM, PVL, and coronary obstruction.

## 4. Surgical Valve Characteristics

**Figure 1:**
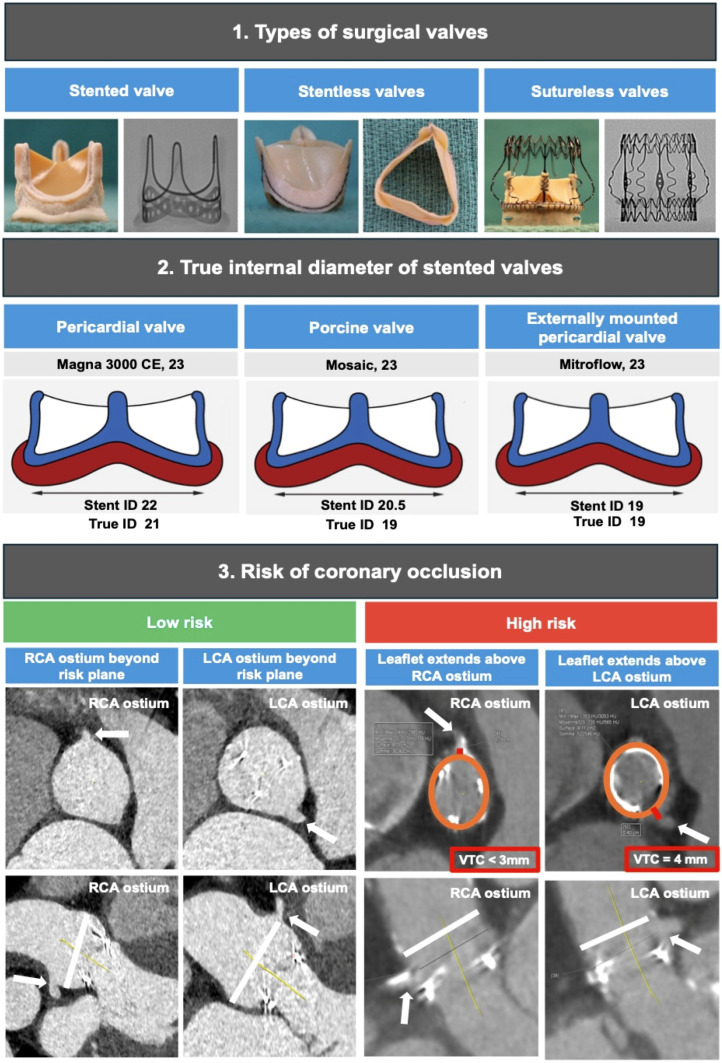
Preprocedural ViV planning; wide arrow indicates both RCA and LCA coronaries ostium; orange circle indicates virtual Evolut Pro + 23 THV; white line indicates upper limit of anticipated neoskirt; red line indicates virtual THV to coronary ostial distance; CE: Carpentier Edwards; ID: internal diameter; LCA: left coronary artery; RCA: right coronary artery.

The procedural success of ViV interventions requires a thorough understanding of the design and mechanisms of bioprosthetic valve failure as well as knowledge of the best available THV options for each patient’s characteristics (Figure 1). Surgical bioprosthetic valves can be stented or stentless according to the presence of a stent frame. Stented valves are the most commonly used and consist of a sewing ring made of fabric at the base of three posts made of metal alloy or plastic material serving as a scaffold to anchor the leaflets. Stented valves are further classified according to the tissue and position of the leaflets into porcine aortic leaflets and bovine pericardial leaflets, and leaflets sutured inside or outside the stent frame [23]. Notably, the latter allows a greater effective orifice area but at the cost of an increased risk of coronary obstruction after ViV [24]. While the labeled size of the prosthesis corresponds to the external diameter of the sewing ring sutured to the native aortic annulus, the true internal diameter is generally smaller and varies depending on the valve type. The radiographic appearance of the valve can also vary across different valve types, including a visible sewing ring and stent posts (e.g., Perimount valve), visible posts tips (e.g., Mosaic valve), barely visible sewing ring (e.g., Epic valve), or radiolucent valve (e.g., Aspire valve).

Stentless valves are characterized by the lack of rigid posts supporting the leaflets, which allows a great orifice area and make this valve type more suitable for small aortic annuli. The sewing ring is sutured to the aortic annulus, and the leaflets, directly to the sinuses of Valsalva. Compared with stented valves which usually suffer from calcific degeneration and stenosis, stentless valves more often degenerate into aortic regurgitation from leaflet malcoaptation or tears. Moreover, stentless valves often lack radiographic markers, making ViV procedures more difficult and associated with worse outcomes with a higher risk of device malposition, coronary obstruction, and PVL [25].

Sutureless valves involve a self- or balloon-expandable stented frame that anchor to the aortic annulus and require few or no sutures. The rapid deployment mechanism allows to minimize cardiopulmonary bypass time while facilitating minimally invasive surgical interventions. With these valve types, there have been concerns around the risk of stent distortion and fatigue, resulting in long-term PVL, as well as post-operative conduction disorders requiring pacemaker implantation [26].

## 5. Technical Aspects

The wire crossing of a bioprosthetic valve does not differ from that of a native aortic valve but may be more challenging. The altered anatomy of the aortic root post surgery, mismatch between the area of the Valsalva sinuses relative to the prosthesis, asymmetrical valve plane, prosthesis misalignment, and bulky valve calcifications can hamper valve crossing during ViV procedures. Furthermore, operators must be aware that accidentally crossing outside the prosthesis frame or through a PVL may occur, with potentially devastating consequences such as valve crushing, embolization, or material entrapment. For this reason, it is important to verify the correct wire pathway through multiple orthogonal views [27].

In rare cases, when the retrograde crossing of severely stenotic bioprosthetic valves cannot be achieved after multiple attempts, an antegrade approach may be considered as a bailout strategy. This involves atrial transseptal puncture to access the left ventricle and cross the bioprosthetic valve with a J-tipped wire. The wire is then snared into the ascending aorta and externalized to form an arterial–venous loop, after which the procedure proceeds in a standard retrograde fashion [28]. Alternatively, a transapical approach could be deemed suitable, especially in patients with hostile vascular accesses or requiring concomitant VIV-transcatheter mitral valve replacement.

The balloon predilatation of degenerative aortic bioprostheses is associated with a significant risk of embolization, stroke, or acute severe aortic regurgitation [29]. However, when balloon predilatation is necessary, such as for severely stenotic and calcified bioprostheses, a small-sized balloon (8–12 mm) is generally preferred.

**Figure 2:**
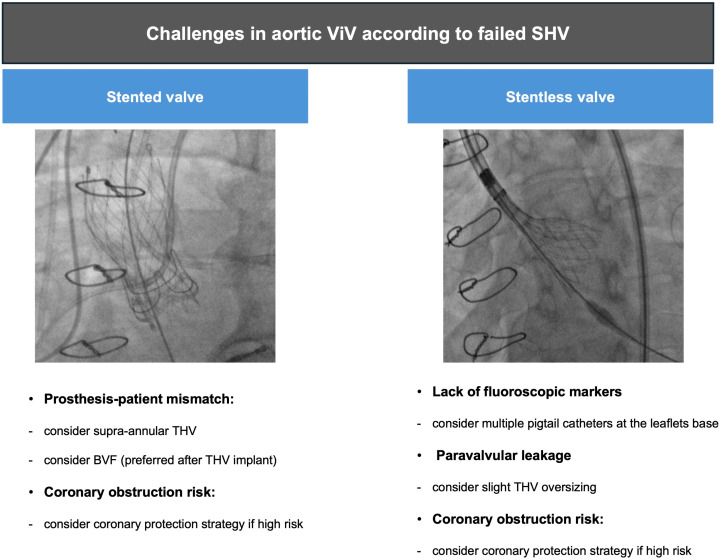
Challenges in aortic ViV-TAVI according to failed SHV; BVF: bioprosthetic valve fracture; SHV: surgical heart valve; THV: transcatheter heart valve; ViV: valve-in-valve.

In case of stentless bioprosthesis or when the sewing ring is not radiopaque, the optimal implantation depth remains challenging, increasing the risk of valve embolization or migration, coronary obstruction, PVL, conduction disturbances, or aortic injury. Figure 2 shows that in such a scenario, two pigtail catheters placed at the base of the leaflets of the degenerated bioprosthesis could be useful to identify the height of the neo-annulus for correct THV deployment. Moreover, implanting a slightly oversized THV may be favored, especially in case of stentless bioprosthesis, to prevent PVL or device embolization.

When THV underexpansion is detected, postdilatation may be performed to improve transvalvular gradients bearing in mind the risk for the excessive expansion of the outflow THV frame [30].

## 6. Commissural Alignment

As surgical valves are generally sutured aligned to the anatomic commissures to preserve aortic root anatomy, commissural alignment remains desirable also during ViV procedures. Preoperative MDCT is used to guide the correct alignment of the THV by identifying the relevant angiographic views for valve deployment. However, while some THVs offer fluoroscopic markers to optimize commissural alignment during the release phase, others do not [31,32].

Bench studies have also shown that commissural alignment improves valve hemodynamics [33]. Valve misalignment may cause a non-physiological transvalvular flow with increased leaflet stress and blood stagnation in the sinus of Valsalva, leading to early valve dysfunction [34]. Commissural misalignment has been associated with twice as much relative THV mean gradient increase >50% from discharge to 1 month (17.6% vs. 8.3%) [35]. Moreover, valve misalignment hampers coronary access after ViV since commissural posts of the THV may create a physical barrier facing coronary ostia [36]. Commissural alignment also influences the risk of sinus sequestration and the ability to perform leaflet modification techniques to prevent coronary obstruction [37].

## 7. Prosthesis–Patient Mismatch

ViV procedures are associated with higher residual transvalvular gradients compared with transcatheter aortic valve intervention (TAVI) of native aortic valves. In this context, the presence of PPM indicates a high discrepancy between the prosthetic valve effective orifice area and the flow demands of the patient. Severe PPM is defined as a valve-effective orifice area indexed to body surface area <0.65 cm^2^/m^2^, and it is strongly associated with increased mortality following ViV [38].

THVs with a supra-annular design are generally preferred in ViV patients with small annuli as they guarantee larger effective orifice areas and better hemodynamics [39,40]. Randomized trials have found no differences in the rates of death or stroke between balloon-expandable and self-expanding THVs at 30 days or 1 year, yet the former have exhibited higher transvalvular gradients [41].

Implantation depth is another major determinant of transvalvular gradient with low THV associated with higher gradients [42,43]. In vitro studies have defined the optimal implantation depth for the CoreValve Evolut (Medtronic, Minneapolis, MN, USA) as 0 to 5 mm, and for the Sapien (Edwards Lifesciences, Irvine, CA, USA), 0 to 2 mm above the ring [44]. Surgical valves present a sewing ring that allows fixation within the native valve annulus or above if supra-annular. The sewing ring represents the narrowest point within a bioprosthetic valve, and, although not always visible on angiography, it can be used as a marker of neo-annulus for anchoring the THV at the optimal implantation depth.

## 8. Valve Fracture

In patients with pre-existing or anticipated PPM, balloon-assisted bioprosthetic valve fracture (or cracking) plays a key role as it allows for an increase in the effective orifice area after ViV with a marked reduction in transvalvular gradients [45,46]. A high-pressure dilatation with noncompliant valvuloplasty balloons, typically sized 1 (up to 3) mm larger than the true internal diameter of the surgical valve, is used to crack the stented frame (Table 2) [47,48]. This intervention is particularly useful in small surgical valves (when the true internal diameter is <20 mm, valve size <22 mm) that carry a substantial risk of a high transvalvular gradient post ViV as compared with larger valves (≥23 mm) [46]. Notably, not all surgical valves are suitable for valve cracking or fracture, including the Medtronic Hancock II and St. Jude Trifecta valves, the latter being modifiable [49]. Online apps (e.g., Cardio Valve [Digimednet BV] or Valve In Valve [RUTSCH]) may help in choosing the appropriate THV based on the true internal diameter of each valve type and size and its fluoroscopic appearance.

Bench tests as well as clinical studies have shown a mean gradient reduction following valve fracture from more than 40 mmHg to 10 mmHg or less [40,50,51]. Low transvalvular gradients were stable at 1-year follow-up and the use of self-expandable THV was shown to be a predictor of lower gradients.

Performing valve fracture, though sometimes necessary, requires careful procedural planning and prompt interventions in case of acute mechanical complications. The best timing to perform valve facture, either before or after THV deployment, has been debated for years. In the first case, there is a considerable risk of severe aortic regurgitation with hemodynamic collapse before THV implantation, whereas the second scenario carries a greater risk of THV embolization and leaflet injury. However, recent data have shown that valve fracture performed after THV implantation offers a superior effective orifice area without significant leaflet injury [52].

## 9. Coronary Occlusion Risk Assessment

Coronary artery obstruction is a rare (<1%) but life-threatening complication of aortic ViV [53]. The placement of a new THV alters the anatomy of the aortic root displacing the leaflets of the previously implanted aortic valve outwards. Displaced leaflets can obstruct the coronary ostia, directly or by sequestering the sinuses of Valsalva at the sinotubular junction. As a result, myocardial ischemia and cardiac arrest may occur.

Registry data have shown that, while ViV procedures carry a three- to four-fold higher risk of coronary obstruction compared with TAVI in a native valve [24], appropriate screening using MDCT coronary angiography can mitigate its occurrence [54].

There are several anatomical and technical parameters influencing the risk of coronary obstruction. The most relevant aspect involves the height of bioprosthetic leaflets and coronary ostia. Leaflet displacement (the so-called neoskirt plane) represents the most important plane to be assessed relative to the coronary plane, as determined by coronary ostia [55].

The design of the previously implanted valve is another key aspect, as stented bioprostheses with externally mounted leaflets can increase the risk of coronary obstruction. Even stentless bioprostheses may present challenges owing to the lack of a rigid frame for THV fixation or lack of fluoroscopic markers, suggesting a potential role for intra-operative transesophageal echocardiography [55]. To optimally predict the risk of coronary obstruction during ViV-TAVI, MDCT imaging may be used to elaborate a virtual image of the final ViV implantation.

**Figure 3:**
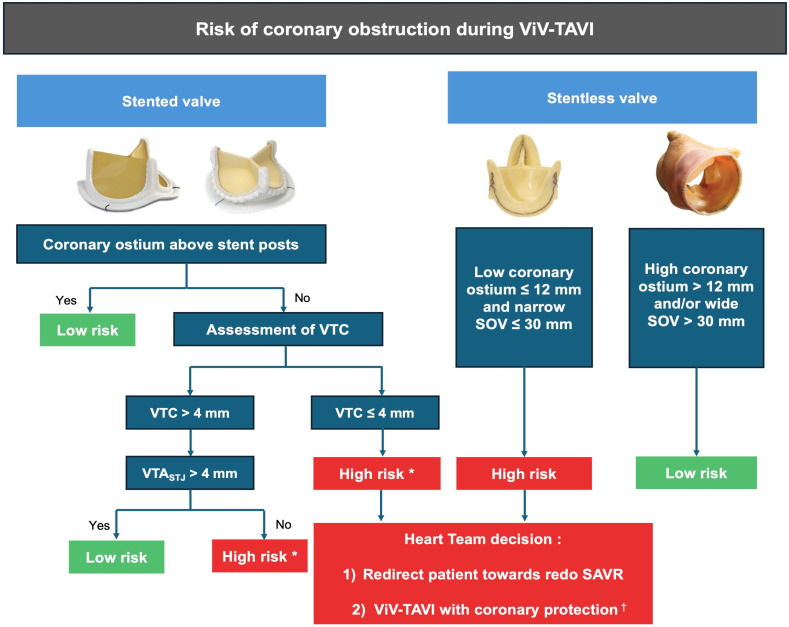
Proposed algorithm for coronary risk obstruction assessment. Especially if bioprosthetic with externally mounted leaflets, ^†^ coronary protection strategies are as follows. Heterotopic snorkel stenting technique: wiring coronary artery and park an undeployed stent to be implanted after THV deployment if coronary flow is impaired. Orthotopic snorkel stenting technique: re-wiring after THV release to have a more physiologic stent implantation through prosthesis valve frame structure. BASILICA: “Bioprosthetic scallop intentional laceration to prevent iatrogenic coronary artery obstruction”, intentional laceration of surgical valve leaflets with electrified guidewire to create communication between sinus and neo-sinus. SAVR: surgical aortic valve replacement; SOV: sinuses of Valsalva; TAVI: transcatheter aortic valve implantation; ViV: valve-in-valve; VTC: virtual transcatheter heart valve to coronary distance; VTA: valve-to-aorta distance, STJ: sinotubular junction. * Especially if bioprosthetic with externally mounted leaflets.

As shown in Figure 3, the distance between the virtual THV and the left or right coronary ostium can help predicting the risk of coronary obstruction. A valve-to-ostium (VTC) distance ≤4 mm has been found in the majority (approximately 90%) of ViV-related coronary occlusions [24]. Meanwhile, with stentless (e.g., Freestyle) or sutureless valves (e.g., Perceval) the risk of coronary obstruction is related to coronary ostia height (≤12 mm) and to the diameter of the sinuses of Valsava (≤30 mm).

## 10. Prevention of Coronary Occlusion

Avoiding THV oversizing, overexpansion, and postdilatation can mitigate the risk associated with outwardly displacing the previous valve leaflets. Performing ViV using a recapturable THV may also be considered, as the device can be repositioned before final release if coronary obstruction occurs.

The commissural alignment of the THV within the previous bioprosthetic valve is also desirable to reduce the risk of coronary obstruction during ViV interventions. Surgical prosthetic valves are generally implanted to guarantee commissural alignment and preserve aortic root anatomy [56]. Conversely, this concept has not been adopted in transcatheter interventions until recently. Only for the most recent THV models (including valves and delivery systems), vendors provide tools to optimize commissural alignment and reduce the risk of coronary obstruction [34].

There are, however, circumstances where the likelihood of coronary obstruction according to MDCT scan parameters is so high that interventional techniques should be adopted to avoid this complication.

**Figure 4:**
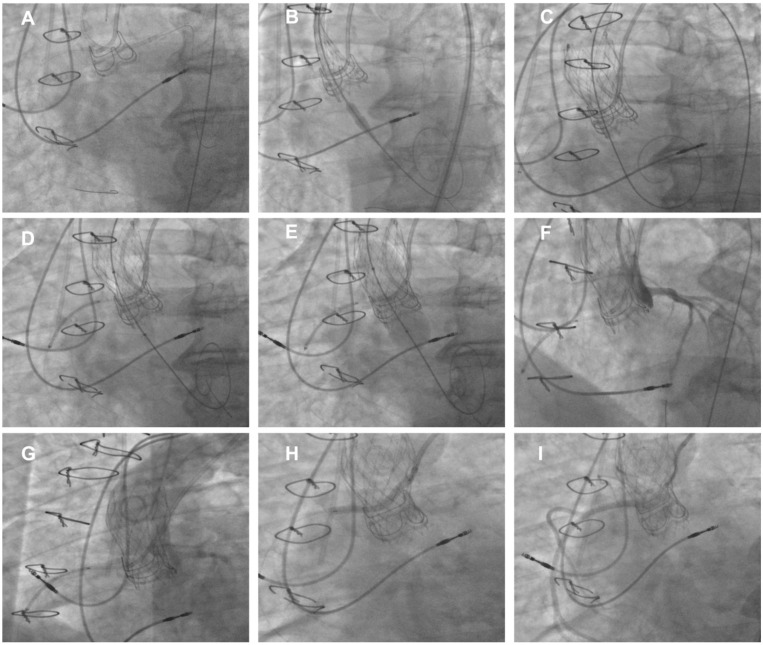
Example of TAV-in-SAV at high risk of coronary obstruction. (**A**) Cannulation of both LCA and RCA using double-radial 6Fr approach. (**B**) Placement of guide extension catheter in RCA before deployment of Evolut Pro+ 23 THV in degenerated CE Magna 3000 21. (**C**) Final deployment of Evolut PRO+ 23 THV. (**D**) Undeployed drug-eluting stent positioned in RCA before post-dilatation. (**E**) Post-dilatation using non-compliant balloon. (**F**) Angiography showing good perfusion of both LCA and left sinus. (**G**) Aortogram showing impaired perfusion of RCA and right sinus while LCA is perfused. (**H**) Pull back and deployment of stent with its proximal portion positioned above neoskirt using chimney technique. (**I**) Final angiography showing good RCA perfusion. SHV: surgical heart valve; THV: transcatheter heart valve; ViV: Valve-in-Valve.

The most used technique is the “chimney stenting” (also called snorkel stenting) technique, which consists of placing a prophylactic guidewire, balloon, or undeployed stent to allow for the prompt treatment of coronary obstruction (Figure 4). It must be noted that chimney stenting is not itself a preventive strategy but rather an acceptable bailout technique used to promptly treat coronary obstruction. Indeed, if coronary blood flow is compromised, the stent is retracted from the left main back to the aorta above the THV upper frame to create a channel, pinned between the displaced leaflets and the aortic root, for coronary perfusion [57]. Notably, cyclic compressions and local vascular perturbations around the entrapped stent may lead to strut deformation and thrombosis. Therefore, chimney stenting should be reserved to patients at low risk for subsequent coronary interventions, since re-access to coronary arteries afterward is challenging, especially in emergency situations. To guarantee a more physiologic stent implantation and ease future re-access to coronary ostia, in the absence of complete coronary obstruction following THV deployment, rewiring and stenting through the THV struts (i.e., orthotopic snorkel stenting) may be attempted [58].

BASILICA (Bioprosthetic or native Aortic Scallop Intentional Laceration to prevent Iatrogenic Coronary Artery obstruction) is an alternative, yet more complex technique, that can be used to prevent coronary obstruction. This procedure involves an electrified wire that is used to split the surgical valve leaflet in two parts such that, when pushed aside by the new THV, it splays in front of the coronary ostium avoiding its occlusion. Prospective investigations have confirmed both the feasibility (procedural success rate of 95%) and safety of the BASILICA technique in patients undergoing TAVI for either bioprosthetic or native aortic valve failure [59]. A retrospective study on 168 patients recently compared these two approaches. Patients undergoing BASILICA had a higher preprocedural risk of coronary obstruction, as indicated by a lower sinotubular junction height (18.2 ± 4.8 mm vs. 14.8 ± 3.4 mm) and diameter (28.2 ± 4.5 vs. 26.8 ± 3.4). However, the rates of periprocedural complications were similar between the BASILICA and chimney tecniques, with clinical success >96% in both groups. At 1-year follow-up, the cumulative incidence of major adverse cardiovascular events was 18.7% in the chimney group and 19.9% in the BASILICA group (*p* = 0.85). A higher rate of acute kidney injury, PVL, and contrast media administration was reported in the chimney group, whereas BASILICA was associated with incremental use of cerebral embolic protection devices [60].

The ShortCut (Pi-Cardia) device is a new tool specifically designed to split bioprosthetic aortic valve leaflets in patients requiring BASILICA during ViV-TAVI. It consists of a splitting element that engages the leaflet in a controlled manner and a positioning arm that protects the splitting element throughout the intervention. The ShortCut procedure is a fluoroscopy- and echocardiography-guided intervention. The dedicated catheter is introduced through a 16 Fr introducer sheath and advanced to the aortic valve over a preshaped guidewire. Once the device is properly aligned at the annular level, the positioning arm is exposed and rotated toward the targeted leaflet; then, the splitting element is activated, engaging the bottom of the leaflet from the ventricular side. The positioning arm situated on the aortic side of the leaflet protects the surrounding aortic tissue from potential injury. The leaflet is then split by gently retracting the catheter. An advantage of this technique is that the splitting of a second leaflet cold be performed by simply rotating the positioning arm and repeating the procedural steps [61,62]. In a pivotal, prospective, single-arm study of 60 patients, leaflet splitting with ShortCut met the primary safety endpoint with no mortality or one disabling stroke (1.7%) within 7 days. Successful leaflet splitting was achieved in 100% of patients, as confirmed by transesophageal echocardiography, and freedom from coronary obstruction was 95% at 30 days [63].

Currently, a few other devices, such as the Splitter device (HVT Medical), for valve leaflet modification by cusp splitting and partial leaflet excision prior to ViV-TAVI, are being developed and tested in vivo [64].

## 11. Valve Thrombosis

Clinical valve thrombosis is common after ViV, particularly in patients not receiving oral anticoagulation [65]. A study with 300 patients with stented (86.3%) and stentless (13.7%) degenerated surgical valves treated by self-expanding (50%), balloon-expandable (49%), and mechanically expanding (1.0%) THVs reported an incidence of clinical valve thrombosis of 7.6%. Of those, fifteen patients (65%) presented with worsening symptoms, and 21 (91%), with transvalvular mean gradient elevation. Protective factors associated with valve thrombosis were oral anticoagulation (OR 0.07) and a larger surgical valve true internal diameter indexed to body surface area (OR 0.54), whereas the Mosaic and Hancock II stented porcine bioprostheses (OR 4.0) were associated with a higher risk of ViV thrombosis [66].

The pathophysiology of valve thrombosis after ViV procedures recognizes different potential mechanisms [67]. The presence of two bioprosthetic surfaces with typically smaller THV sizes and higher gradients may favor a hypercoagulable environment [68]. Valve asymmetric deployment, which may occur during ViV procedures, can lead to suboptimal cusp coaptation and excursion and further increase thromboembolic risk. Moreover, the presence of narrow neo-sinuses and non-commissural alignment can enhance blood stasis while reducing washout time. Blood washout within the neo-sinuses is generally altered after TAVI, and ViV further contributes to blood flow perturbations. Factors influencing washout time include THV implantation level (sub-, intra-, or supra-annular), expansion (over-, under-expanded), and coronary flow dynamics. Most studies agree that supra-annular THV positioning results in a decreased washout time. Intentional lacerations of the native aortic leaflets have been associated with significant improvement in neo-sinuses washout time following TAVI [69,70].

**Figure 5:**
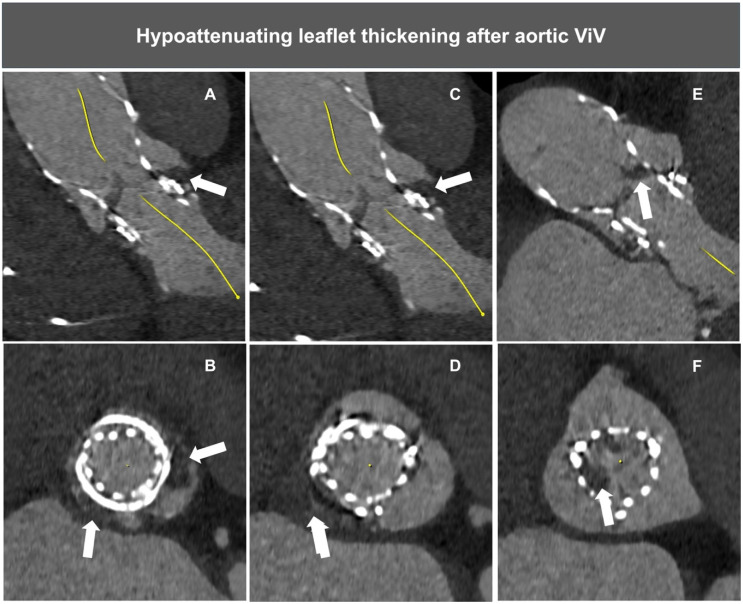
Example of HALT after aortic ViV. Wide arrow indicates hypoattenuating leaflet thickening (HALT). (**A**–**D**) Hypodensity corresponding to partial thrombosis of sinuses of Valsalva. (**E**,**F**) HALT of right and non-coronary THV leaflets.

Valve thrombosis can be detected using transthoracic echocardiography or contrast MDCT, the latter being more accurate in identifying the signs of thrombus formation (Figure 5). As ViV patients represent a subset at a relatively high thrombotic risk, systematic cardiac CT follow-up assessment may allow for the prompt identification and treatment of this condition.

The GALILEO trial demonstrated that in patients without an established indication for oral anticoagulation after successful TAVI, a low dose of rivaroxaban, 10 mg, was associated with a higher risk of death or thromboembolic complications and a higher risk of bleeding than an antiplatelet-based strategy [71]. In the GALILEO-4D substudy, however, low-dose rivaroxaban was more effective in preventing subclinical leaflet motion abnormalities [72]. Similarly, in the ATLANTIS-4D-CT study, apixaban, compared with antiplatelet therapy, reduced subclinical obstructive valve thrombosis [73]. Unfortunately, only about 5% of patients enrolled in GALILEO and ATLANTIS were treated with ViV-TAVI, and subgroup analyses were largely underpowered to extrapolate conclusions in this cohort. Dedicated studies are therefore needed to address the pharmacological management of ViV interventions where a more intensive antithrombotic approach may theoretically shift the risk–benefit ratio of oral anticoagulation toward a greater net clinical benefit.

## 12. Cerebral Protection

Periprocedural stroke is a rare but life-threatening complication of TAVI, and its occurrence has been reported to be <1.5% during ViV interventions [74]. Interestingly, registry data suggest higher rates of strokes (3.4%) during ViV, particularly when performed with the BASILICA technique to prevent acute coronary obstruction [75]. While the recent PROTECTED TAVR trial showed that the use of cerebral protection devices does not have a significant effect on the incidence of periprocedural stroke overall, disabling strokes were reduced from 1.3% to 0.5% [76]. In this setting, cerebral protection devices during ViV may be a valuable tool for stroke risk prevention, with observational studies suggesting a marked benefit associated with their use [75]. Further prospective investigations are needed to confirm these preliminary data.

## 13. Conclusions

ViV-TAVI represents a significant advancement in the field of interventional cardiology, offering a less invasive treatment option for patients considered at intermediate to high risk for repeat surgical valve replacement. Utilizing minimally invasive transcatheter techniques, ViV has demonstrated promising results, including symptom relief, improved hemodynamics, and enhanced quality of life for patients with degenerated bioprosthetic aortic valves. Nevertheless, patient selection, preprocedural planning, operator expertise, and new device iterations remain of paramount importance for ensuring optimal results and reducing complications rates. As technology continues to evolve and experience accumulates, the optimization of ViV interventions holds the potential to broaden treatment indications and potentially transform the management of aortic valve disease across various patient populations.

## Figures and Tables

**Table 1:** Outcomes of patients treated with aortic ViV in large registries.

Study	*n*	Age (Years)	STS Score	30-Day Mortality	1-Year Mortality	30-Day Stroke	1-Year Stroke	30-Day HF/Repeated Admission	1-Year HF Admission
Dvir 2014 [18]	459	77.6	9.8%	7.6%	16.9%	1.7%	-	-	-
Tuzcu 2018 [17]	1150	79	6.9%	2.9%	11.7%	1.7%	3.2%	2.4%	9.2%
Hahn 2022 [16]	365	78.9	9.1%	2.7%	11.8%	2.5%	4.5%	2.5%	4.6%

STS: The Society of Thoracic Surgeons Predicted Risk of Operative Mortality score; HF: heart failure.

**Table 2:** Summary of bench testing of high-pressure balloon inflation to fracture stent frame of surgical valves.

Manufacture/Brand	Valve Size (s)	Fracture Possible	Fracture Pressure
St. Jude Trifecta	19–21 mm	no	-
St. Jude Biocor Epic	21 mm	yes	8 atm
Medtronic Mosaic	19–21 mm	yes	10 atm
Medtronic Hancock II	21 mm	no	-
Sorin Mitroflow	19–21 mm	yes	12 atm
Edwards Magna Ease	19–21 mm	yes	18 atm
Edwards Magna	19–21 mm	yes	24 atm

Adapted from Alle KB et. Ann Thorac Surg. 2017 Nov; 104 (5): 1501–1508 [46].
